# Decoding the Interactions Regulating the Active State Mechanics of Eukaryotic Protein Kinases

**DOI:** 10.1371/journal.pbio.2000127

**Published:** 2016-11-30

**Authors:** Hiruy S. Meharena, Xiaorui Fan, Lalima G. Ahuja, Malik M. Keshwani, Christopher L. McClendon, Angela M. Chen, Joseph A. Adams, Susan S. Taylor

**Affiliations:** 1 Biomedical Sciences, University of California, San Diego, La Jolla, California, United States of America; 2 Department of Chemistry and Biochemistry, University of California, San Diego, La Jolla, California, United States of America; 3 Department of Pharmacology, University of California, San Diego, La Jolla, California, United States of America; 4 Skaggs School of Pharmacy and Pharmaceutical Sciences, University of California, San Diego, La Jolla, California, United States of America; University of Minnesota, United States of America

## Abstract

Eukaryotic protein kinases regulate most cellular functions by phosphorylating targeted protein substrates through a highly conserved catalytic core. In the active state, the catalytic core oscillates between open, intermediate, and closed conformations. Currently, the intramolecular interactions that regulate the active state mechanics are not well understood. Here, using cAMP-dependent protein kinase as a representative model coupled with biochemical, biophysical, and computational techniques, we define a set of highly conserved electrostatic and hydrophobic interactions working harmoniously to regulate these mechanics. These include the previously identified salt bridge between a lysine from the β3-strand and a glutamate from the αC-helix as well as an electrostatic interaction between the phosphorylated activation loop and αC-helix and an ensemble of hydrophobic residues of the Regulatory spine and Shell. Moreover, for over three decades it was thought that the highly conserved β3-lysine was essential for phosphoryl transfer, but our findings show that the β3-lysine is not required for phosphoryl transfer but is essential for the active state mechanics.

## Author Summary

Eukaryotic protein kinases (EPKs) regulate over a third of the human proteome by transferring the γ-phosphate from adenosine triphosphate (ATP) to a protein substrate in a process known as protein phosphorylation. Biochemical and biophysical studies have shown that EPKs undergo multiconformational rearrangements in which the catalytic core is oscillating between open, intermediate, and closed conformations when active. Presently, the intramolecular interactions that regulate this dynamic process are not well understood. In this paper, we show how a set of conserved electrostatic and hydrophobic interactions harmoniously regulate the active state mechanics. The electrostatic interactions involve the highly conserved salt bridge between the lysine from subdomain-II and glutamate from subdomain-III as well as an interaction between the activation loop and αC-helix. The hydrophobic interactions include the nonlinear motifs known as the Regulatory spine and Shell that traverse both lobes of the catalytic core. Furthermore, our findings show that the highly conserved “catalytic lysine” is not directly required for phosphoryl transfer but rather serves as a hub that aligns and positions the dynamic core elements required for catalysis.

## Introduction

Eukaryotic protein kinases (EPKs) were first discovered in 1943, and their functional role in phosphorylation was elucidated in 1956 [1–3]. In 1968, cAMP-dependent protein kinase (PKA) was the second protein kinase to be discovered, and in 1969, it was shown that phosphorylation is not tissue- or species-specific [4]. PKA has since served as the prototype for our understanding of EPK structure and function. EPKs are ubiquitously expressed in all eukaryotes, and approximately 2% of the human genome encodes for EPKs [5,6]. EPKs are involved in most biological processes and have been associated with numerous human diseases, making EPKs key candidates for therapeutic intervention [7].

**Fig 1:**
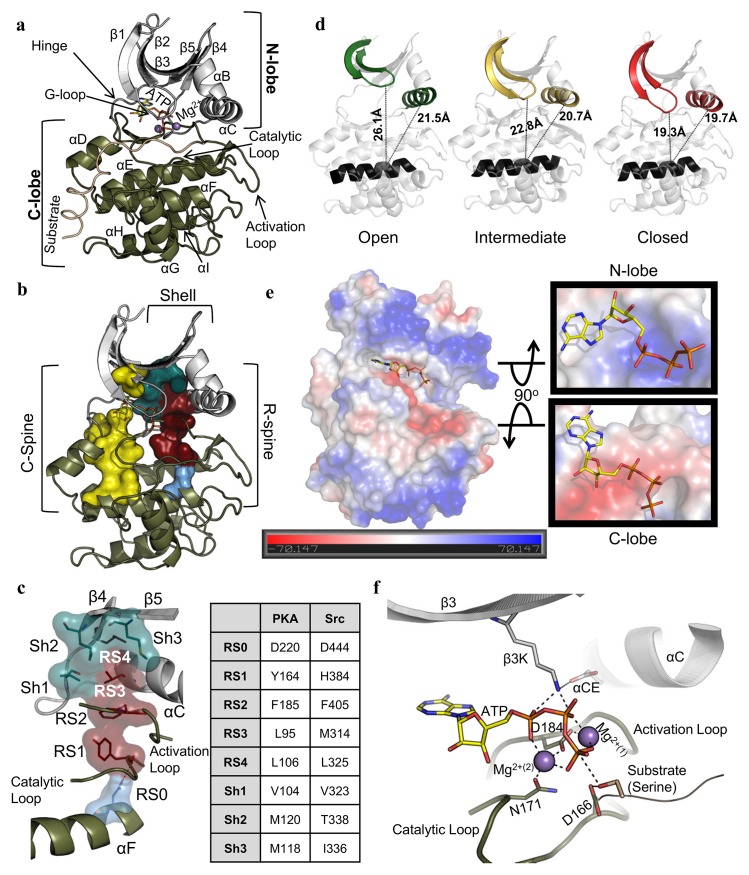
Global architecture of the EPK core. **A.** The structural core mapped on the catalytic subunit of PKA (PDBID:1ATP). The N-lobe (grey) is mostly composed of β-sheets and the C-lobe (olive) is mostly α-helical. The active site is between these lobes and ATP binds with two magnesium ions. **B.** R-spine (maroon), Shell residues (teal), and C-spine (yellow). **C.** Orientation of the specific residues of the R-spine (maroon) (labeled as RS1 from the catalytic loop [olive], RS2 from the activation loop [olive], RS3 from the αC-helix [grey], and RS4 from the β4 strand [grey], which is anchored to the αF-helix [olive] by RS0 [light blue]), Shell (teal) (Sh1 from the αC-β4 loop [grey], Sh2 and Sh3 from the β5-strand [grey]), and a table showing the R-spine and Shell residues of PKA and Src as representatives of serine/threonine and tyrosine kinases, respectively. **D.** A structural representation of the open (green [PDBID:4NTS]), intermediate (yellow [PDBID:1BKX]), and closed (red [PDBID:1ATP]) conformations of PKA measured by the distance from the center of the αF-helix (A223-Cα) to the G-loop (S53-Cα) and αC-Helix (H87-Cα). **E.** Electrostatic potential of the catalytic core shows that the N-lobe active site (top right) is mostly positive, whereas the C-lobe active site (bottom right) is highly negative. **F.** Active site interactions before phosphoryl transfer with ATP and substrate bound in the active site (PDBID:4DG2).

EPKs share a highly conserved catalytic core that mediates the transfer of the γ-phosphate of adenosine triphosphate (ATP) to a protein substrate [8]. Structurally, the core consists of two lobes, the N-lobe and C-lobe (Fig 1A) [9]. Within the core there are two nonlinear hydrophobic motifs known as the Catalytic (C)-spine and the Regulatory (R)-spine that span both lobes (Fig 1B) [10]. The R-spine includes two residues from the C-lobe (RS1 [Y164] from the **Y/H**rd motif in the catalytic loop [CL] and RS2 [F185] from the d**F**g motif in the activation loop [AL]) and two from the N-lobe (RS3 [L95] from the αC-helix and RS4 [L106] from the β4-strand) (Fig 1C). The R-spine is anchored to the αF-helix through a highly conserved aspartate (RS0 [D220]) [11]. The R-spine is supported by an ensemble of conserved hydrophobic residues referred to as the Shell (Sh1 [V104] and Sh2 [gatekeeper, M120] from the αC-β4 loop and Sh3 [M118] from the β5-strand) [11,12]. EPKs are typically in equilibrium between the inactive and active states in which the R-spine is disassembled and assembled, respectively [11,13]. After the assembly of the R-spine, the active state of an EPK toggles between open, intermediate, and closed conformations as it traverses the catalytic cycle [9,14–16]. The transition from the open to the closed conformation is initiated by the binding of ATP, which transitions the core from the open to an intermediate conformation (Fig 1D). This transition is primarily driven by the interaction of the adenine ring of ATP with the C-spine and the hinge region, a process that fuses the N- and C-lobe portions of the C-spine [17]. The binding of a substrate allows the final transition from the intermediate to the closed conformation [18].

The C-lobe portion of the active site cleft of most EPKs has a negatively charged electrostatic surface and thus requires two magnesium ions (Mg^2+^) to fully neutralize the negative charge of the phosphates (Fig 1E) [15]. These Mg^2+^ ions bind to two conserved residues from the C-lobe—an aspartate (D184 in PKA) from the DFG motif in the AL and an asparagine (N171) from the CL—to position the γ-phosphate of ATP for phosphoryl transfer (Fig 1F) [19]. The α/β-phosphates of ATP interact with the highly conserved lysine residue in the β3-strand (β3K [K72]), which forms a salt bridge with a conserved glutamate from the αC-helix (αCE [E91]) [20,21]. It was initially assumed that β3K was required for ATP binding and the salt bridge with αCE was required for stabilizing β3K. However, subsequent biochemical data showed that mutation of these residues abolished catalytic activity but had no impact on ATP binding, and therefore the precise role of these residues in catalysis remained unclear [22,23]. The Glycine-rich Loop (G-loop) of the N-lobe—which interacts with ATP, substrate, and the C-lobe—seals the active site chamber in the closed conformation. When the substrate (serine, threonine, or tyrosine) is positioned by the conserved aspartate from the HRD motif in the CL (D166), phosphoryl transfer is achieved [24]. Finally, the phosphorylated substrate and adenosine diphosphate (ADP) are released and the EPK transitions back to an open conformation, where it is poised to bind another ATP molecule to repeat this process [25].

Although the end points of the phosphoryl transfer process have been elucidated and each step in the catalytic cycle has been captured in a crystal lattice, the interactions that facilitate the catalytic cycle remain unclear. Using an *Escherichia coli* expression system, site-directed mutagenesis, western blotting, a radioactive phosphoryl transfer assay, molecular dynamics (MD) simulations, molecular modeling, and a thermal shift assay, we have deciphered the interactions required for the mechanics of the catalytic cycle as well as phosphoryl transfer. Using PKA as a model system, we show that the β3K-αCE salt bridge is required for connecting the αC-helix with the β-sheet region of the N-lobe during the catalytic cycle and that in the absence of the salt bridge, the mechanics can be restored by strengthening the hydrophobicity of the R-spine and Shell residues. The positive charge of β3K is required for neutralizing the negative charge on αCE, and in the absence of β3K, our MD simulation data indicates that the negative charge of αCE interacts with the Mg^2+^ ions, causing misalignment of the γ-phosphate, which inhibits phosphoryl transfer. Surprisingly, although mutating β3K on its own is inactive, removing both β3K and αCE simultaneously partially restores catalytic activity.

## Results

### Is the Salt Bridge Required for Positioning β3K or the αC-Helix?

**Fig 2:**
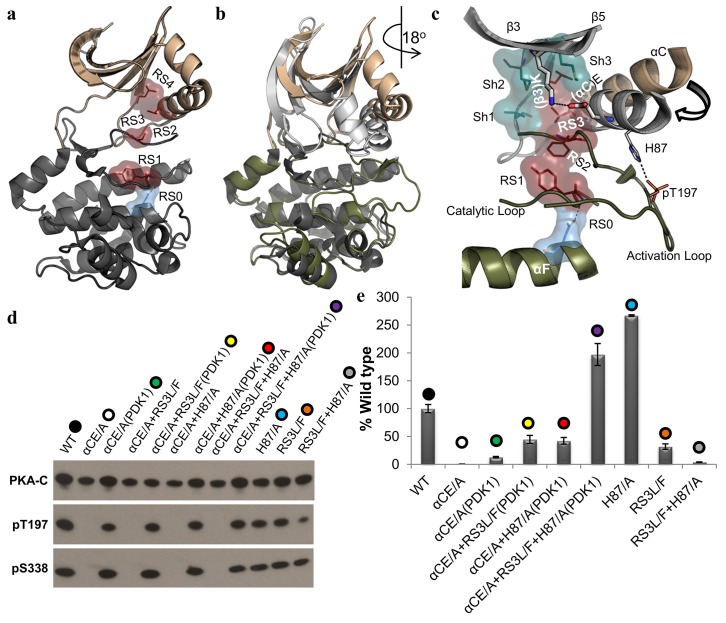
Role of the salt bridge in catalytic activity. **A.** Structural representation of the inactive form of PKA, in which the R-spine (maroon) is disassembled, and the Shell residues (teal). **B.** A structural comparison of the active (grey and olive [PDBID:1ATP]) and inactive (tan and black [PDBID:4DFY]) PKA structures showing the twisted conformation of the inactive conformation. **C.** Structural representation of the required transition of the αC-helix from the inactive (tan) to the active (grey) conformation. The electrostatic interactions between the αC-helix (H87) and the AL (pT197) and the salt bridge between the αC-helix (αCE) and β3-strand (β3K) as well as the assembled R-spine and Shell residues are present in the active closed conformation. **D.** Western blot of the different mutants showing the expression (PKA-C) and autophosphorylation state of the AL and C-tail (pT197 and pS338, respectively). **E.** Level of radioactive phosphoryl transfer of the different mutants as compared to the WT-C data represents the mean and standard deviation shown in S1 Table.

PKA has two phosphorylation sites: a *trans*-autophosphorylation site on the AL (T197) and a *cis*-autophosphorylation site that lies outside of the core on the C-terminal tail (S338) [26]. Phosphorylation of the AL has been shown to be the mechanism for regulating the transition from the inactive to active state [27]. The dephosphorylated AL in PKA induces disassembly of the R-spine as well as a twisting of the N-lobe away from the C-lobe (Fig 2A and 2B) [28]. The dephosphorylated form maintains ~5% catalytic activity, which suggests that the dephosphorylated form of PKA is still oscillating between the inactive and active states (Fig 2C). We hypothesize that in the absence of the salt bridge, the position of the αC-helix is not regulated with the β-sheet region of the N-lobe and thus will not transition into the active conformation with the rest of the N-lobe. As previously shown, the αCE to alanine (αCE/A) mutation cannot undergo either *cis* or *trans* autophosphorylation (Fig 2D) [29], and a radioactive phosphoryl transfer assay with a small peptide (kemptide) shows less than 0.5% catalytic activity in comparison to the wild-type PKA catalytic subunit (WT-C) (Fig 2E and S1 Table). To facilitate the synchronization of the αC-helix through the assembly of the R-spine, we coexpressed αCE/A with 3-phosphoinositide-dependent protein kinase 1 (PDK1) [30]. PDK1 *trans*-phosphorylates T197 and thus nucleates a network of interactions in the C-lobe, which facilitates assembly of the R-spine. When αCE/A is coexpressed with PDK1 (αCE/A[PDK1]), it is now also able to undergo *cis*-autophosphorylation of S338 (pS338) as well as an enhanced radioactive phosphoryl transfer of ~13%. We then decided to further stabilize the αC-helix in the active conformation by introducing a bulkier hydrophobic phenylalanine at RS3 (RS3L/F). The nonphosphorylated form of αCE/A+RS3L/F remains inactive, but αCE/A+RS3L/F coexpressed with PDK1 has ~45% catalytic activity as compared to WT-C. Therefore, the salt bridge is required for stabilizing the αC-helix, and introducing a bulky residue into the R-spine partially rescues the absence of the salt bridge. These results show that the salt bridge is required for positioning the αC-helix.

### Is the Salt Bridge Required for Opening and Closing?

We propose that in the absence of the salt bridge (αCE/A), the catalytic activity is diminished because the αC-helix remains anchored to the AL through the pT197-H87 interaction. To address this, we replaced H87 with an alanine (H87/A) in the salt bridge–deficient mutant (αCE/A+H87/A). This enhanced the catalytic activity to over 40% when coexpressed with PDK1 but remained inactive when expressed independently (Fig 2D and 2E). We then further stabilized the αC-helix in the αCE/A+H87/A mutant through the RS3L/F mutation (αCE/A+RS3L/F+H87/A). This mutant had ~200% catalytic activity when coexpressed with PDK1. A previous study showed that the H87/A mutant had a 2- to 3-fold increase in catalytic activity, which correlated with our results in which H87/A had 267% catalytic activity as compared to the WT-C [31]. The equilibrium of closing and opening as well as the stability of the R-spine is a delicate balance between hydrophobic and electrostatic interactions, as the introduction of RS3L/F or RS3L/F+H87/A into the WT-C reduces the catalytic efficiency to ~30% and ~4%, respectively. These results indicate that the salt bridge is involved in both opening and closing and also demonstrates that the interaction of H87 with the AL phosphorylation site acts as an anchor that slows down opening of the active site cleft. These results also show that the salt bridge is required for linking the αC-helix with the β-sheet region in the catalytic cycle process.

### Is the αC-helix Regulated by Hydrophobic or Electrostatic Interactions?

**Fig 3:**
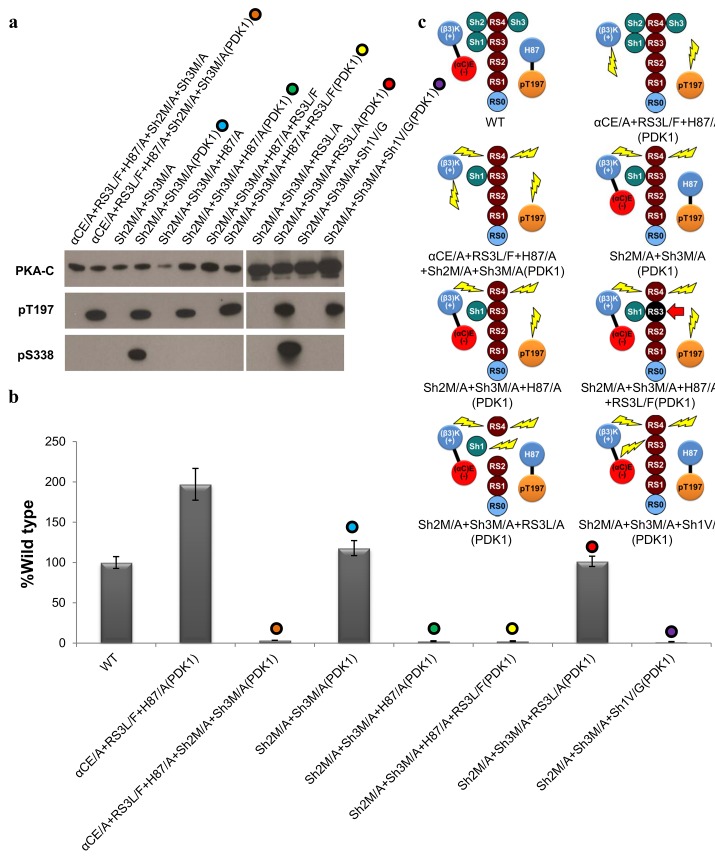
Hydrophobic versus Electrostatic interactions. **A.** Western blot of the different mutants showing the expression (PKA-C) and autophosphorylation state of the AL and C-tail (pT197 and pS338, respectively). **B.** Level of radioactive phosphoryl transfer of the different mutants as compared to the WT-C data represents the mean and standard deviation shown in S1 Table. **C.** Illustrated representation of the mutants shown in panel **B.**

The αCE/A+RS3L/F+H87/A(PDK1) mutant is ~2-fold more active than WT-C in the absence of the salt bridge and the pT197-H87 electrostatic interactions (Fig 2D and 2E). To address if the catalytic activity of this mutant is regulated by the hydrophobic interactions of the Shell residues, we mutated Sh2 (gatekeeper) and Sh3 from the β5-strand simultaneously to alanine (αCE/A+RS3L/F+H87/A+Sh2M/A+Sh3M/A). This mutant was inactive when expressed independently and when coexpressed with PDK1, indicating that a stable R-spine, independent of the surrounding Shell residues, is not sufficient for catalytic activity (Fig 3A, 3B and 3C). Next, to understand if catalytic activity can be achieved with just the electrostatic interactions, we introduced the Sh2M/A+Sh3M/A mutations into the WT-C. Previously, we have shown that this mutant is catalytically inactive when expressed independently [11], but when the mutant was coexpressed with PDK1 to introduce the pT197-H87 interaction to the already existing salt bridge (Sh2M/A+Sh3M/A[PDK1]), the catalytic activity was rescued to ~115%. Removal of the pT197-H87 interaction through the H87/A mutation (Sh2M/A+Sh3M/A+H87/A) abolished catalytic activity when expressed independently or coexpressed with PDK1. In contrast to the H87/A mutant, which is regulated by the R-spine and Shell, these results indicate that in the absence of the Shell residues, the pT197-H87 interaction is required for regulating the αC-helix. To address whether the R-spine stabilizing mutant (RS3L/F) can rescue catalytic activity in the absence of the Shell residues and the pT197-H87 interaction, we generated another mutant (Sh2M/A+Sh3M/A+H87/A+RS3L/F). In this case, the enhanced hydrophobicity of the R-spine was insufficient to rescue catalytic activity when expressed independently or coexpressed with PDK1. This indicates that the R-spine requires the support of the shell residues to regulate the αC-helix. We then hypothesized that in the shell-deficient mutant, the αC-helix is positioned by the two electrostatic interactions (salt bridge and pT197-H87) independent of the R-spine. To test this, we mutated RS3 to an alanine (RS3L/A) in the Shell-deficient mutant (Sh2M/A+Sh3M/A+RS3L/A). This mutant maintained catalytic activity comparable to WT-C (~101%) when coexpressed with PDK1. Finally, to address whether the presence of all Shell residues is required for catalytic activity, a triple Shell mutant Sh2M/A+Sh3M/A+Sh3V/G was generated. Catalytic activity in the absence of all Shell residues is abolished when expressed independently or coexpressed with PDK1. Our previous studies showed that Sh1 is a crucial residue that is uniquely positioned to interact with both the N- and C-lobes, and any alterations to this residue diminishes catalytic activity [11]. These results collectively indicate that two systems synergistically regulate catalytic activity: the hydrophobic ensemble (R-spine + Shell) and the two electrostatic pairs (salt bridge + pT197-H87). While we have teased apart the individual interactions regulating the active state mechanics, in their natural states both the electrostatic and hydrophobic systems work in harmony to regulate catalytic activity.

### What Are the Consequences of Altering β3K?

Previous studies indicated that mutation of β3K to alanine (β3K/A), methionine (β3K/M), histidine (β3K/H), and arginine (β3K/R) have no catalytic activity [22,32–34]. To understand if the loss of catalytic activity of these mutants was due to the loss of the salt bridge, we first coexpressed these mutants with PDK1 (S1A and S1B Fig). The β3K/A, β3K/M, and β3K/H mutants remained inactive for *cis*-autophosphorylation, *trans*-autophosphorylation, and for steady-state catalysis. In contrast, the β3K/R mutant was able to undergo *cis*-autophosphorylation but had very low levels of steady-state radioactive phosphoryl transfer activity (~6%). In an attempt to enhance the catalytic activity, we simultaneously introduced the R-spine stabilizing mutation (RS3L/F) and removed the interaction between the αC-helix and the AL (H87/A). These mutations did not yield an increase in catalytic activity for any of the mutants. This led us to believe that β3K had more functional roles than just participating in the formation of a salt bridge with αCE.

### Is β3K Required for Orienting the N-lobe for Catalysis?

**Fig 4:**
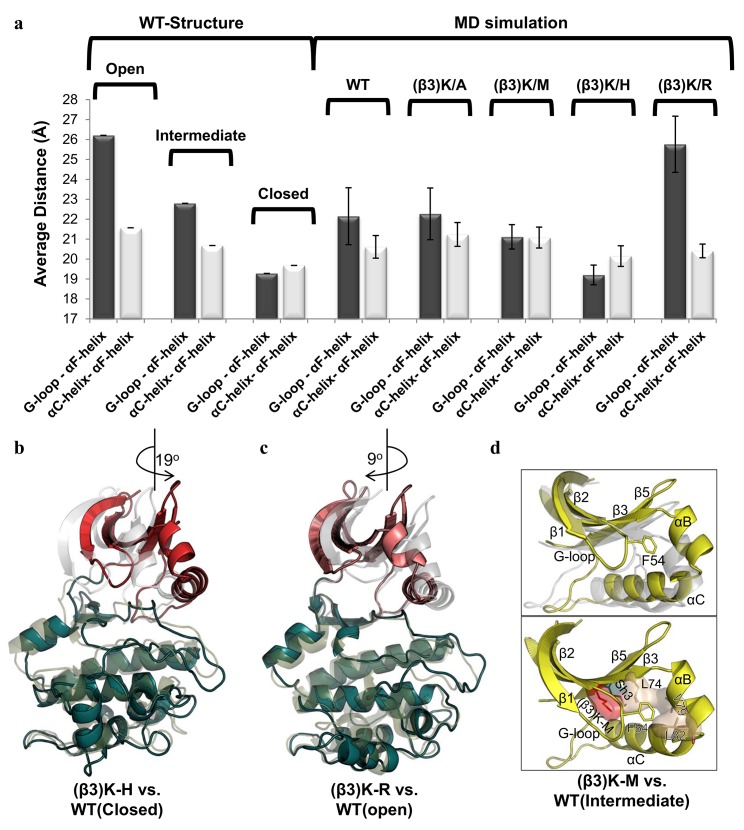
Global intermediate conformation of the WT-C and β3K mutants. **A.** Average distance from the center of the αF-helix (A223-Cα) to the G-loop (S53-Cα) and αC-Helix (H87-Cα); data shown in S2 Table. **B.** Structural representation of the unique G-loop conformation observed in β3K/M compared to the WT-C (top) and the specific interactions of the hydrophobic clamp unique to the β3K/M mutant (bottom). **C.** Structural representation of twisted conformation of the β3K/H mutant (red and teal) in comparison to the WT-C closed conformation (grey and olive). **D.** Structural representation of the more open intermediate conformation of β3K/R (pink and teal) in comparison to the WT-C open conformation (grey and olive).

To elucidate the role of β3K, we ran MD simulations of the WT-C, β3K/A, β3K/M, β3K/H, and β3K/R mutants with the AL phosphorylated (pT197). The MD simulations were started with the closed (ATP + substrate-bound) structure (PDBID:3FJQ) [35]. We then removed the substrate to simulate the intermediate ATP-bound conformation for WT-C, β3K/A, β3K/M, β3K/ H, and β3K/R to search for any conformational changes that could explain the inactivation of these mutants. We used principal component analysis (PCA) to elucidate the largest collective motions. The first principal component corresponded to the active site opening, and the second principal component corresponded to interlobe twist. With datasets of ~700 ns in length, we were able to visualize the conformational change from the closed to intermediate state for the WT-C and all the mutants (S2A and S2B Fig). The WT-C MD simulation mimicked an intermediate structure, with an average distance of 22.2 Å between the G-loop and αF-helix and 20.6 Å between the αC-helix and αF-helix (Figs 1D and 4A, S2C Fig, and S2 Table). The PCA of β3K/A and β3K/M shows significant Cα conformational overlap with WT-C. The β3K/A MD simulation data showed an intermediate conformation, with an average distance of 22.3 Å between the G-loop and αF-helix and 21.2 Å between the αC-helix and αF-helix. The MD simulation for the β3K/M mutant has an average distance of 21.1 Å between the αC-helix and αF-helix, whereas the average distance between the G-loop and αF-helix was slightly more closed, with an average distance of 21.1 Å as compared to the WT-C. In contrast, for β3K/H and β3K/R, the αB-helix and the αC-helix showed substantial changes over the trajectory, reflecting an altered twist in the N-lobe as compared to WT-C. The β3K/H simulation shows a more closed conformation, with an average distance of 19.2 Å between the G-loop and αF-helix but a greater average distance of 20.2 Å between the αC-helix and αF-helix. This is due to the twisting of the N-lobe by ~19^o^ counter-clockwise from the C-lobe (Fig 4B). This twisted conformation resembles the dephosphorylated conformation of PKA, but β3K/H is more closed and maintains an assembled R-spine (S3 Fig). Finally, the β3K/R simulation shows a more open conformation, with an average distance of 25.8 Å between the G-loop and αF-helix and an average distance of 20.4 Å between the αC-helix and αF-helix because of a clockwise rotation (~9^o^) between the N- and C-lobes (Fig 4C). Previous structural studies of the β3K/R mutation of the extracellular signal–regulated protein kinase 2 (ERK2) shows a similar ATP-bound intermediate conformation, with a distance of 26 Å from the G-loop to the αF-helix and 20.4 Å between the αC-helix and αF-helix [22]. Thus, alternative side chains at β3K tend to yield an altered relative orientation of the N-lobe and C-lobe, while removal of this sidechain β3K/A retains a WT-C interlobal orientation. These MD simulation data indicate that β3K is involved in orienting the N-lobe.

We used the Kullback–Leibler divergence as an information-theoretic approach to quantify changes in the φ, ψ, and χ dihedral distributions for each mutant versus the WT-C and to assess the statistical significance of these changes [36]. Comparing the β3K/A and β3K/M mutants to WT-C, the Kullback–Leibler divergence highlighted most the dihedral perturbations in the DFG-motif of the activation loop, the αC-helix, the CL, the αH-αI loop, and the αI-helix (S2D Fig and S3 Table). Additionally, β3K/M showed more substantial perturbations in the G-loop. The hydrophobic nature of β3K/M induces a conformational change in the side chain of β3K/M through a hydrophobic interaction with Sh3 (Fig 4B). This conformational change induces a side chain flip of the neighboring leucine (L74), which results in the formation of a hydrophobic clamp with the aid of two hydrophobic residues from the αB-helix (V79 and L82). A conserved aromatic residue from the G-loop (F54) [37] docks into this pocket and attains gauche^+^ χ1 and χ2 torsion angles as compared to gauche^-^ and gauche^+^ (χ1 and χ2 torsion angles, respectively) in the WT-C (S4 Fig). In the β3K/H and β3K/R mutants, the αC-helix and DFG-motif of the AL showed milder perturbation but had the largest perturbation at the end of the αE-helix, and β3K/R specifically had perturbations on the G-loop. Overall, the β3K/A and β3K/M showed larger perturbations in the dihedrals of the αC-helix, CL, and AL compared with β3K/H and β3K/R.

### What Is the Electrostatic Impact of Removing β3K?

**Fig 5:**
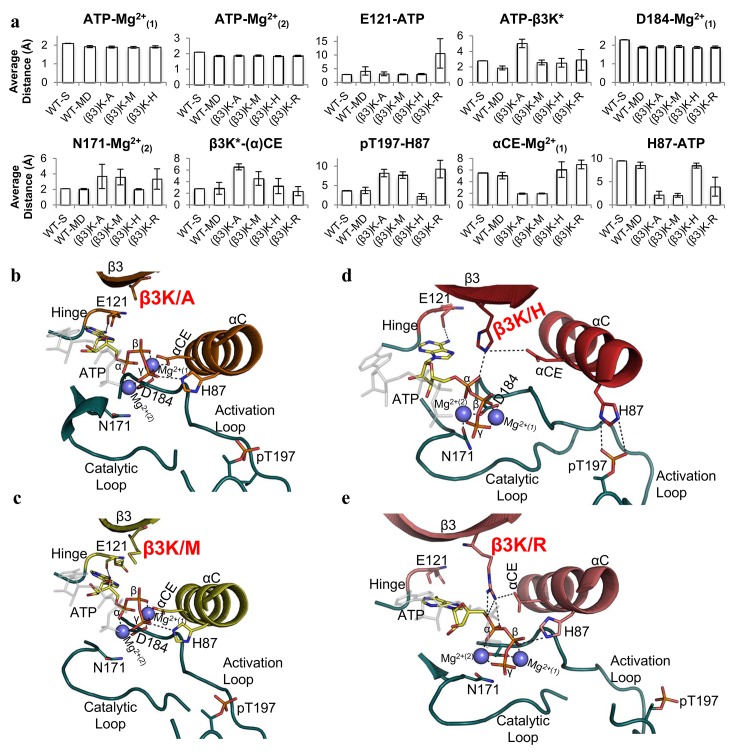
Active site conformation of the WT-C and β3K mutants. **A.** Average distance (data shown in S4 Table) measurements capturing the essential interactions required for catalytic activity. Structural representation of the active site interactions of **B.** β3K/A mutant, **C.** β3K/M mutant, **D.** β3K/H mutant, and **E.** β3K/R mutant aligned to the αF-helix of the WT-C (grey) intermediate conformation.

The MD simulation of the WT-C (WT-MD) shows that the intermediate conformation maintains all the required interactions of the active site observed in the WT-C structure (WT-S) (Figs 1F and 5A, S5 Fig, and S4 Table). In the β3K/A and β3K/M mutants, which lack the positive charge of β3K, there is an unoccupied negative charge on αCE that interacts with Mg^2+^_(1)_, which is constitutively bound to ATP. This interaction induces an interaction between the γ-phosphate of ATP and H87 that stabilizes ATP in a nonproductive binding conformation (Fig 5A–5C and S5 and S6 Figs). In the MD simulations of these mutants, the misalignment of the γ-phosphate results in the dissociation of N171 from Mg^2+^_(2)_, with an average distance of 3.7 Å and 3.6 Å in β3K/A and β3K/M, respectively (cutoff distance ~2.3 Å) [38]. The β3K/H mutant maintains all the required interactions in the active site, but the twisting of the lobes induces a nonproductive conformation (Fig 5A and 5D). The β3K/R mutant simulations show that ATP is dislodged from the hinge (E121) and assumes multiple conformations within the active site (Fig 5A and 5E and S5 and S6 Figs). We observe that in the first 250 ns of the trajectory, the average distance between β3K/R and the ribose/α-phosphate oxygen is 2.8 Å, whereas in the WT-C the average distance is 4.7 Å. The interaction of β3K/R with the ribose/α-phosphate oxygen results in the unhinging and flexibility of ATP from its catalytically competent conformation (S6 Fig). This unhinging allows ATP to sample conformations near the αC-helix, promoting the interaction of H87 with the β-phosphate of ATP (3.9 Å), and as a result the interaction between N171 and Mg^2+^_(2)_ is broken (3.3 Å). Previous structural studies of the kinase suppressor of Ras (KSR) (a pseudokinase with a β3K/R mutation) as well as the ERK2-β3K/R mutant have shown defects in the binding of ATP [22,39]. In the β3K/R simulation, ATP oscillates between the hinged and unhinged conformations, which could explain the ~6% and ~5% basal catalytic activity observed in β3K/R(PDK1) and ERK2-β3K/R mutants, respectively [22]. These results suggest that that the β3K is involved in structurally poising the active site cleft for catalysis.

### Is It Possible to Rescue Catalytic Activity in the (β3)K Mutants?

**Fig 6:**
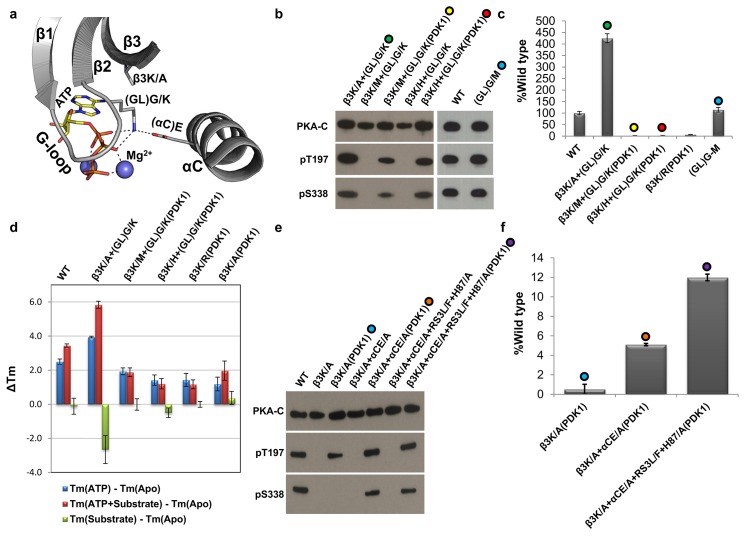
Rescue of catalytic activity through a G-loop mutation. **A.** Molecular modeling of β3K/A+(GL)G-K mutant. **B.** Western blot of the different mutants showing the expression (PKA-C) and autophosphorylation state of the AL and C-tail (pT197 and pS338, respectively). **C.** Level of radioactive phosphoryl transfer of the different mutants as compared to the WT-C data represents the mean and standard deviation shown in S1 Table. **D.** Thermal shift assay capturing the conformational changes depicting the transition from the open to the intermediate and from the intermediate to the closed conformation through the addition of ATP and ATP + substrate. Data shown in S5 Table. **E.** Western blot of the different mutants showing the expression (PKA-C) and autophosphorylation state of the AL and C-tail (pT197 and pS338, respectively). **F.** Level of radioactive phosphoryl transfer of the different mutants as compared to the WT-C data represents the mean and standard deviation shown in S1 Table.

To address this question, we introduced a lysine on the last glycine of the G-loop (G55), which is a natural variant first identified in WNK (with no lysine) kinase [40,41]. We first introduced the G-loop glycine to lysine ([GL]G/K) mutation into β3K/A (β3K/A+[GL]G/K). This mutant had ~400% the catalytic activity of WT-C and was independent of PDK1 (Fig 6A, 6B and 6C). We then introduced the (GL)G/K mutation into β3K/M (β3K/M+(GL)G/K) and β3K/H (β3K/H+(GL)G/K) to see if this mutation can unclamp the G-loop and untwist the lobes, respectively. Both of these mutants had no catalytic activity when expressed independently. When coexpressed with PDK1, both β3K/M+(GL)G/K and β3K/H+(GL)G/K were able to undergo *cis*-autophosphorylation (pS338) but had very low levels of steady-state catalytic activity (1.84% and 2.5%, respectively). To make sure that this inactivity is not induced by a steric clash between two bulky residues on β3K and (GL)G, we introduced a (GL)G/M mutation in WT-C, and we observed a catalytic activity ~114% of WT-C (Fig 6B and 6C). The β3K/A+(GL)G/K results show that it is possible to rescue the catalytic activity by repositioning the positive lysine onto the G-loop.

### Are the β3K Mutants Deficient in Closing?

We hypothesized that the β3K/M+(GL)G-K(PDK1), β3K/H+(GL)G-K(PDK1), and β3K/R(PDK1) were catalytically inefficient because of their inability to attain a productive closed conformation as a result of the hydrophobic clamp in β3K/M, the twisting of the lobes in β3K/H, and the misalignment of ATP in β3K/R. Previous studies of PKA showed that the conformational changes induced by the binding of ATP and ATP + substrate result in a shift in thermal stability [42]. We thus utilized a thermal shift assay to capture these conformational changes for the various β3K mutants. The addition of ATP induced an increase in the melting temperature (ΔTm) of 2.5°C, 3.9°C, 1.9°C, 1.4°C, and 1.4°C in the WT-C, β3K/A+(GL)G/K, β3K/M+(GL)G/K(PDK1), β3K/H+(GL)G/K(PDK1), and β3K/R(PDK1), respectively (Fig 6D and S5 Table). This correlates with previous studies that have shown that mutations of β3K did not impact ATP binding [43], but the β3K mutants are less effective at transitioning from the open to the intermediate conformation. To capture the ATP + substrate-bound closed conformation, we used a pseudosubstrate inhibitor (IP20)—which has an alanine instead of a serine or threonine at the P-site—which prevents phosphoryl transfer and thus traps a stable closed conformation. The addition of ATP + substrate to the WT-C and β3K/A+(GL)G/K mutant yields a ΔTm of 3.4°C and 5.8°C, respectively, whereas the addition of ATP + pseudosubstrate to β3K/M+(GL)G/K(PDK1), β3K/H+(GL)G/K(PDK1), and β3K/R(PDK1) had no significant ΔTm as compared to the ATP-bound state. This indicates that these three mutants are unlikely to achieve a perfectly closed conformation. Finally, pseudosubstrate binding to the Apo conformation had no conformational change to the WT-C, β3K/M+(GL)G/K(PDK1), β3K/H+(GL)G/K(PDK1), or β3K/R(PDK1), with the exception β3K/A+(GL)G/K, which had a ΔTm of –2.5°C. These results indicate that the β3K/A+(GL)G/K is 4-fold more active than the WT-C because it undergoes a more stable transition in response to the binding of ATP and ATP + pseudosubstrate, while the binding of pseudosubstrate in the absence of ATP destabilizes the protein, making this mutant highly efficient. These results show that β3K is required for the conformational transition from the open to the intermediate and from the intermediate to the closed conformation.

### Is β3K Required for Phosphoryl Transfer?

Although β3K/A+PDK1 is unable to facilitate steady-state phosphoryl transfer, it still undergoes a conformational change from open to intermediate (ΔTm = 1.2°C) and from intermediate to closed state with the addition ATP and ATP + pseudosubstrate (ΔTm = 2°C), even though the enhanced stability is not as robust as the WT-C or β3K/A+G55/K (Fig 6C). The MD simulation data suggested that the absence of the positive charge of β3K/A leaves the negative charge on αCE free to interact with the Mg^2+^, causing a structurally nonproductive active site pocket (Fig 5A–5C). To correct for this, we mutated both β3K and αCE to an alanine (β3K/A+αCE/A) to remove both the positive and negative charge, respectively. When β3K/A+αCE/A is expressed independently, neither T197 nor S338 are phosphorylated, but after coexpression with PDK1—(β3K/A+αCE/A[PDK1])—which allows for *trans*-autophosphorylation of T197, we observe that S338 can now also become *cis*-autophosphorylated, and the radioactive phosphoryl goes up to ~5% of WT-C as compared to β3K/A+PDK1, which has no catalytic activity. Next, we strengthened the R-spine through RS3L/F and removed the interaction between the αC-helix and AL (H87/A) to enhance flexibility of the αC-helix (β3K/A+αCE/A+RS3L/F+H87/A) (Fig 6D and 6E). When this mutant was coexpressed with PDK1, it was able to *cis*-autophosphorylate S338, and the steady-state catalysis is further enhanced to ~12% of WT-C. Even though this mutant has an increased rate of phosphoryl transfer, it is still more than an order of magnitude less than WT-C and αCE/A+RS3L/F+H87/A(PDK1), indicating that the β3K is a crucial element for achieving a functional active state. Although β3K is not required for phosphoryl transfer, it is nevertheless a structural ATP sensor that facilitates the active state mechanics by coordinating the transition of the β-sheet region of the N-lobe and linking this region with the αC-helix. These results collectively show that β3K is not required for phosphoryl transfer.

## Discussion

According to our spine hypothesis and reinforced by NMR studies [10,14,16], the binding of the adenine ring of ATP drives the transition of the EPK core from the open to the intermediate conformation by assembling the C-spine. This transition also coordinates the electrostatic interaction of β3K with αCE and the α/β-phosphates of ATP. Breaking the β3K–αCE salt bridge by mutating αCE to an alanine destabilizes the active conformation that is associated with the assembled spine, where it cannot undergo *cis-*autophosphorylation, *trans*-autophosphoryaltion, or steady-state catalysis. The assembly of the R-spine, however, can be partially restored by coexpressing αCE/A with PDK1, which introduces an interaction between the phosphorylated activation loop (pT197) and H87 from the αC-Helix. These concerted effects associated with phosphorylation of the activation loop allow the αC-helix to be positioned in an “active” state where it can now transition from the open to the intermediate and closed conformations. Our results show, however, that even though αCE/A+PDK1 regained catalytic activity, the steady-state catalysis remained low, most likely because of the inability of the EPK core to unhinge the αC-helix from the AL. To ameliorate this deficiency, we mutated H87 to an alanine so that the αC-helix was no longer electrostatically anchored to the AL. The αCE/A+H87A(PDK1) double mutant had a ~50% increase in steady-state catalysis, confirming that assembly of the R-spine by phosphorylation is due to the linking of the AL phosphate with the HRD motif and not to the interaction with H87. Following AL phosphorylation, the active conformation is not stabilized independently of H87; instead, H87 appears to negatively regulate the steady-state phosphoryl transfer by impeding opening of the catalytic cleft. These results indicate that the salt bridge is required for closing as well as opening. In the absence of the salt bridge as well as the electrostatic interaction between the activation loop and the αC-helix, the active state mechanics can be regulated through the R-spine and Shell. By enhancing the hydrophobicity of RS3 from a leucine to a phenylalanine, we were able to connect the β-sheet region and the αC-helix through the hydrophobic interaction between the Sh2 and Sh3 from the β-sheet region and RS3 from the αC-helix. The αCE/A+H87A+RS3L/F(PDK1) is ~2-fold more catalytically active than the WT-C. These results indicate that the active state mechanics of EPKs are harmoniously regulated by both electrostatic and hydrophobic interactions.

Our results also show that β3K is required for sensing ATP and enabling the transition of the EPK core from the open to the intermediate conformation, and any mutation of β3K significantly reduces this conformational shift. The inability of the β3K/A mutant to transition from the open to the intermediate conformation is rescued to ~4-fold of WT-C by introducing a lysine on the G-loop, which mimics WNK-kinase. In contrast, the other β3K mutants were unable to achieve similar catalytic activities because of the clamped G-loop conformation induced by β3K/M, the inactive-like clockwise twisting of the lobes in the shorter β3K/H mutant, and the counter-clockwise twisting of the N-lobe in the bulkier β3K/R mutant, based on our MD simulation data. For more than a quarter century, it was believed that β3K was directly involved in phosphoryl transfer by positioning the γ-phosphate of ATP [13]; however, our MD simulation data indicated that in the absence of the positive charge on β3K, the free negative charge on the partnering αCE starts interacting with the Mg^2+^, resulting in the delocalization of the γ-phosphate. By removing both of the charges on β3K/A and αCE/A, we were able to partially rescue the catalytic activity that was lost in the single β3K/A mutant. In conjunction with previous findings, our results unambiguously indicate that β3K is not required for binding or positioning ATP and is not essential for phosphoryl transfer. To make sure that β3K is not only involved in connecting the αC-helix with the β-sheet region but also sensing ATP, we introduced the enhanced R-spine + Shell system into the double mutant (β3K/A+ αCE/A+H87/A+RS3L/F), and the catalytic activity was only rescued to ~12% of the WT-C as compared to αCE/A+H87/A+RS3L/F, which is ~2-fold more active than WT-C, indicating that β3K is required for connecting the different regions of the N-lobe in the presence ATP but not required for phosphoryl transfer.

Currently, any variation of β3K is a key feature used for identifying pseudokinases [5], but our results indicate that there are other mechanisms that can compensate for these variations. We therefore believe that an in-depth analysis of all the catalytic components is required prior to definitively labeling an EPK as a pseudokinase. Furthermore, EPK mutations have been reported in numerous diseases, but one of the major challenges is differentiating between causative and noncausative mutations without extensive experimental analysis [44]. This study opens a new avenue of predicting disease-causing mutations that do not generate a constitutively active or inactive EPK but enhance the rate of catalytic activity once the EPK has been activated. It is also currently known that EPKs have noncatalytic roles in biological processes, and β3K mutants are widely utilized to decipher these noncanonical functions [45]. Our findings show that these mutations should be designed with caution, as the conformational differences between the different mutants may interfere with these noncanonical functions as well.

## Materials and Methods

### Site-Directed Mutagenesis

QuikChange II site-directed mutagenesis kit (Agilent Technologies) was used to introduce mutations. The following primers were used to introduce mutations:

E91A Forward

5′′-ATC GAG CAC ACT CTG AAT GCG AAG CGC ATC CTG CAG GCC-3′

E91A Reverse

5′-GGC CTG CAG GAT GCG CTT CGC ATT CAG AGT GTG CTC GAT-3′

L95F Forward

5′-AAG CGC ATC TTG CAG GCC GTC AAC TTC CCG TTC CTG GTC-3′

L95F Reverse

5′-GAC GGC CTG CAA GAT GCG CTT CTC ATT CAG AGT GTG CTC-3′

L95A Forward

5′-CTG AAT GAG AAG CGC ATC GCG CAG GCC GTC AAC TTC CCG-3′

L95A Reverse

5′-CGG GAA GTT GAC GGC CTG CGC GAT GCG CTT CTC ATT CAG-3′

H87A Forward

5′-AAG CTA AAG CAG ATC GAG GCG ACT CTG AAT GAG AAG CGC-3′

H87A Reverse

5′-GCG CTT CTC ATT CAG AGT CGC CTC GAT CTG CTT TAG CTT-3′

E91A/H87A Forward

5′-ATC GAG GCC ACT CTG AAT GCG AAG CGC ATC CTG CAG GCC-3′

E91A/H87A Reverse

5′-GGC CTG CAG GAT GCG CTT CGC ATT CAG AGT GGC CTC GAT-3′

L95F/E91A Forward

5′-CTG AAT GCG AAG CGC ATC TTC CAG GCC GTC AAC TTC CCG-3′

L95F/E91A Reverse

5′-CGG GAA GTT GAC GGC CTG GAA GAT GCG CTT CGC ATT CAG-3′

M118A/M120A Forward

5′-TCA AAC CTG TAC GCG GTC GCG GAG TAT GTA GCT GGT GGC-3′

M118A/M120A Reverse

5′-GCC ACC AGC TAC ATA CTC CGC GAC CGC GTA CAG GTT TGA-3′

V104G Forward

5′-GTC AAC TTC CCG TTC CTG GGG AAA CTT GAA TTC TCC TTC-3′

V104G Reverse

5′-GAA GGA GAA TTC AAG TTT CCC CAG GAA CGG GAA GTT GAC-3′

K72A Forward

5′-TAC GCC ATG GCG ATC TTA GAC AAG CAG AAG GTG GTG AAG-3′

K72A Reverse

5′-GTC TAA GAT CGC CAT GGC GTA GTG GTT CCC ACT CTC CTT-3′

K72M Forward

5′-TAC GCC ATG ATG ATC TTA GAC AAG CAG AAG GTG GTG AAG-3′

K72M Reverse

5′-GTC TAA GAT CAT CAT GGC GTA GTG GTT CCC ACT CTC CTT-3′

K72H Forward

5′-TAC GCC ATG CAC ATC TTA GAC AAG CAG AAG GTG GTG AAG-3′

K72H Reverse

5′-GTC TAA GAT GTG CAT GG CGT AGT GGT TCC CAC TCT CCTT-3′

K72R Forward

5′-GGG AAC CAC TAC GCC ATG AGG ATC TTA GAC AAG CAG AAG-3′

K72R Reverse

5′-CTT CTG CTT GTC TAA GAT CCT CAT GGC GTA GTG GTT CCC-3′

G55K Forward

5′-GGC TCC TTT AAG CGA GTG ATG CTG GTG AAG CAC AAG GAG-3′

G55K Reverse

5′-CAT CAC TCG CTT AAA GGA GCC GGT GCC AAG GGT CTT GAT-3′

G55M Forward

5′-CTT GGC ACC GGC TCC TTT ATG CGA GTG ATG CTG GTG AAG-3′

G55M Reverse

5′-CTT CAC CAG CAT CAC TCG CAT AAA GGA GCC GGT GCC AAG-3′

### Protein Expression and Purification

The 6X-Histidine–tagged WT and mutants containing PKA in pET15b as well as mutants were coexpressed with GST-tagged PDK1 in BL21 (DE3). Cultures were grown at 37°C to an A600 of ∼0.6 and induced with 0.5 mM IPTG. The cultures were allowed to grow overnight at 18°C before being harvested. The pellet was resuspended in lysis buffer (50 mM KH2PO4, 20 mM Tris-HCl, 100 mM NaCl, 5 mM β-mercaptoethanol, pH 8.0) and lysed using a microfluidizer (Microfluidics) at 18,000 p.s.i. The cells were clarified by centrifugation at 18,000 rpm at 4°C for 45 min in a Beckman JA20 rotor, and the supernatant was incubated with Probond Nickel resin (Invitrogen) overnight at 4°C using gravity. The resin was washed twice (10x bed volume) with the lysis buffer and twice with using two different concentrations of imidazole in the wash buffer (50 mM KH2PO4, 20 mM Tris-HCl, 150 mM NaCl, 50mM/100mM imidazole, and 5 mM β-mercaptoethanol, pH 7). A 250 mM imidazole elution buffer was used to elute the His-tagged protein. Samples containing the most protein were dialyzed overnight into 20 mm KH2PO4, 20 mm KCl, and 2.5 mm DTT, pH 6.5 and then loaded onto a prepacked Mono S 10/10 (GE Healthcare) cation exchange column equilibrated in the same buffer. The protein was eluted with an equilibration buffer containing a KCl gradient ranging from 20 mM to 1 M. The Expression of PKA was confirmed using PKA C-subunit antibodies from BD Biosciences, the phosphorylation state of the activation loop was confirmed using a polyclonal pT197 antibody from Invitrogen, and the phosphorylation of the C-tail terminal was confirmed using a pS338 antibody from ThermoFisher Scientific after purification. Separate western blots were run for each antibody staining.

### Protein Folding

The His6-tagged murine Cα-subunit of PKA containing catalytically inactive mutants were coexpressed with GST-tagged PDK1 in BL21 (DE3). PDK1 requires a properly folded PKA to phosphorylate PKA on T197 [46]. Cultures were grown at 37°C to an A600 of ∼0.6 and induced with 0.5 mM IPTG. The cultures were allowed to grow overnight at 18°C before harvesting. The expression of PKA was confirmed using PKA C-subunit antibodies from BD Biosciences, and the phosphorylation state of the activation loop was confirmed using a polyclonal pT197 antibody from Invitrogen after purification.

### Radioactive Phosphoryl Transfer Assay

The kinetics were carried out with a common reaction mix containing 50 mM MOPS pH 7.4, 1mM Kemptide (only phosphorylated by PKA) [47], 10mM MgCl_2_, 1mM ATP, and ^32^ γP radiolabelled ATP (specific activity 500–1,000 cpm/pmol) in a final volume of 20 μL. The reaction was initiated by addition of PKA, with final concentration of 50 nM in a volume of 10 μl to 10 μl of the reaction mix described above. The reaction was carried out as an end-point assay with 3 min as the fixed time, and at the end point the reaction was quenched with 90 μl of 30% acetic acid. 50 μl of the quenched reaction was then spotted on p81 phosphocellulose paper, washed three times for 5 min each with 5% phosphoric acid, and finally washed with acetone (1X), air dried, and counted on a liquid scintillation counter. The background counts were subtracted from the experimental time points and plotted to compare their activities. The wild-type protein was used as positive control and β3K/A mutant as the negative control. Each reaction was carried out in triplicates, and data plotted are mean percent of wild type ± standard error. The plots were made using Microsoft Excel.

### Structural Representations and Molecular Modeling

All images, electrostatic potential, and structural distance calculations were done using the PyMOL molecular graphics system (Schrödinger, LLC). For molecular modeling, mutants were generated in PyMOL and were then submitted to the YASARA minimization server [48]. Comparative figures were aligned by the αF-helix unless specifically stated.

### Molecular Dynamics System Preparation, Simulations, and Analysis

Starting with a high-resolution structure of PKA in the closed conformation with ATP and two Mn2+ ions (PDBID:3FJQ), the Protein Local Optimization Program was used to pre-process the PDB file and optimize or mutate and optimize the side chain at position β3K, and then the structure was imported into Maestro (Schrödinger, LLC). The remainder of the preparation was done using the procedure described previously [49].

The program AMBER14 was used for the initial energy minimization, heating, and equilibration steps using the pmemd.cuda module with hydrogen mass repartitioning. Each system was energy minimized, first with position restraints on the protein and ATP/Mg2+ and then without restraints. Constant volume simulations using particle mesh Ewald with a 10 Å cutoff for range-limited interactions were used. Molecular dynamics with a 2 fs time-step were performed to heat the systems from 0 K to 300 K linearly over 5.0 ns, with 10.0 kcal/mol/Å position restraints on the protein and ATP/Mg2+. Temperature was maintained by the Langevin thermostat, with a collision frequency of 1.0 ps-1. Then, constant pressure dynamics were performed with isotropic position scaling, first with position restraints for 100 ps and a relaxation time of 360 ps and then without position restraints for 2.4 ns.

Production runs in the NVT ensemble were conducted using AMBER14 with particle mesh Ewald using an 8 Å cutoff for range-limited interactions. Temperature was maintained by the Langevin thermostat at 300 K, with a collision frequency of 2.0 ps-1. A time-step of 2 fs was used, and snapshots were collected every 100 ps for the first 2 ns, and then the time-step was changed to 4 fs for the remainder of the production run, with snapshots collected every 240 ps. Triplicate simulations were completed for each system, with simulation times for each replicate (including equilibration) of 1.13 microseconds (WT-C, β3K/A, β3K/M), 712 ns (β3K/H), and 794 ns (β3K/R). The distances and dihedral angles were calculated for a single simulation using Gromacs or VMD, and the data was exported into Microsoft Excel, where traces, line graphs, histograms, average distances, and standard deviations were calculated for the first 712 ns unless specified. Density distribution histograms were generated for the all triplicate simulations.

### Principle Component Analysis

To visualize global backbone conformational differences between wild type and each mutant, we performed principal components analysis. To define a reduced coordinate system, we lumped together the C-alpha coordinates for one trajectory for each mutant into a combined dataset and calculated the first two principal components, which alone contributed 35% to the total variation in the C-alpha coordinates. Then, we projected the trajectories of C-alpha atoms of wild-type and mutant datasets onto these principal components. Using one long simulation for each mutant allows us to visualize global motions from the beginning of the simulation (colored red), through the middle (colored white), and to the end (colored blue). Visually, our first two principal components were consistent with those identified by Masterson et al. for wild-type PKA [16]: the first principal component corresponded to the active site opening, and the second principal component corresponded to interlobe twist.

### Kullback–Leibler Divergence Analysis

For each mutant β3K/A, β3K/H, β3K/M, and β3K/R, the triplicate MD simulations were compared to the WT-C using the previously reported Kullback–Leibler divergence analysis [50,51], available in the MutInf package [52] through Stanford’s SimTK. We used the Kullback–Leibler divergence as an information-theoretic approach to quantify changes in the φ, ψ, and χ dihedral distributions for each mutant versus the wild type and asses the statistical significance of these changes. A similar approach was used to identify residues that might act as control points for conformational transitions [36]. By mapping Kullback–Leibler divergence values as colors onto structures, we provide an easy-to-visualize picture of which residues are perturbed the most by each mutation. For each dihedral, 30° bins were used to highlight more rotamer population shifts and less subtle changes. The minimum of the numbers of snapshots from wild-type and mutant datasets was used in cases in which the number of snapshots differed. Residues that did not show statistically significant dihedral population shifts upon mutation are colored white, while statistically significant perturbations go from dark blue (weakest) to cyan, teal, green, yellow, orange, and then red (strongest).

### Thermal Shift Assay

The WT-C and different β3K mutants were expressed and purified as previously described [28]. Differential scanning fluorimetry was used to estimate the shift in melting temperature of the WT-C and mutants upon binding of ATP and/or the substrate (peptide inhibitor IP20). 2.0 μM (4.5 μg in 50 μl) of protein was used in 25 mM HEPES pH 7.0, 100 mM NaCl, 10 mM MgCl_2_, and 1 mM DTT [42]. Duplicate samples were prepared for Apo protein, protein with ATP, protein with ATP + substrate, and protein with substrate. ATP and substrate were used at final concentrations of 1 mM and 25 μM, respectively. Samples were incubated on ice for 10 min prior to adding 5x working concentration of SYPRO orange fluorescent dye (Invitrogen). All samples were read on a CFX96 Touch Real-Time PCR Detection System (Bio-Rad). Temperature scan mode was used to heat the samples from 10 ^o^C to 90 ^o^C at a rate of 1 ^o^C/min, with a halt time of 1 min after every 1 ^o^C. Fluorescence from the protein-bound SYPRO orange was read using FAM (492 nm) and ROX (610 nm) filters for excitation and emission, respectively [53]. Sigmoidal curves were obtained according to the Boltzmann equation:

y=LL+(UL−LL)/(1+exp[(Tm−x)/a])

where LL and UL are the upper and lower limits of fluorescent intensities and a is the slope of the curve within the melting temperature Tm. Tm values for thermal unfolding could be estimated from the first derivative curves as fitted by the CFX96 machine. For more precise Tm values, curves were fitted to a sigmoid equation to estimate its inflexion point using the nonlinear regression module of GraphPad software. Data was exported to Microsoft Excel, where histograms where generated.

## Supporting Information

S1 Fig Catalytic activity of the different β3K mutants and WT-C. **A.** Western blot of the different mutants showing the expression (PKA-C) and autophosphorylation state of the AL and C-tail (pT197 and pS338, respectively). **B.** Level of radioactive phosphoryl transfer of the different mutants as compared to the WT-C data represents the mean and standard deviation shown in S1 Table.
(TIF)

S2 Fig MD simulation analysis elucidating the global profile changes observed in WT-C and mutants. **A.** The trajectory of the C-alpha atoms for one simulation for each construct were projected onto the first two principal components of the combined C-alpha coordinates and are shown in tube representation colored by timestep, where red shows the beginning of the trajectory, white shows the middle, and blue shows the end. **B.** Eigenvalues of the covariance matrix. **C.** Distance calculations from the center of the αF-helix (A223-Cα) to the G-loop (S53-Cα) and αC-Helix (H87-Cα). **D.** Mapping Kullback–Leibler divergence values as colors onto structures of WT-C and mutants.
(TIF)

S3 Fig R-spine and Shell conformations of the β3K mutants. Structures of β3K/A (orange and teal), β3K/M (yellow and teal), β3K/H (red and teal), and β3K/R (pink and teal) are aligned with the WT-C (grey and olive) intermediate conformation.
(TIF)

S4 Fig Dihedral torsion angles of F54 from the G-loop in WT-C and the different β3K mutants. φ, Ψ, χ1, and χ2 torsion angles for F54 from the G-loop are represented as black, red, blue, and green lines, respectively.
(TIF)

S5 Fig Trajectory distance calculations elucidating the active site profile. Distances were calculated between the highly conserved interactions of the active site for the WT-C and the different mutants.
(TIF)

S6 Fig ATP positioning of the different β3K mutants. In comparison to the WT-C, structures of β3K/A (orange and teal), β3K/M (yellow and teal), β3K/H (red and teal), and β3K/R (pink and teal) are aligned with the WT-C (grey and olive) intermediate conformation. The different ATP conformations illustrated for the β3K/R mutant are snapshots at approximately 220 ns, 500 ns, and 700ns.
(TIF)

S1 Table Radioactive phosphoryl transfer assay. (PDF)

S2 Table Average distances from the αF-helix (A223) to the G-loop (S53) and αC-helix (H87) of MD simulations. (PDF)

S3 Table Kullback–Leibler divergence values for each mutant versus WT-C. (PDF)

S4 Table Average distances elucidating the active site profile of MD simulations. (PDF)

S5 Table Melting temperature (Tm) and thermal stability shift (ΔTm). (PDF)

## Abbreviations

αCE: αC-helix
αCE/A: αCE to alanine
β3K: β3-strand
β3K/A: β3K to alanine
β3K/H: β3K to histidine
β3K/M: β3K to methionine
β3K/R: β3K to arginine
AL: activation loop
ADP: adenosine diphosphate
CL: catalytic loop
C-spine: catalytic spine
EPK: eukaryotic protein kinase
ERK2: extracellular signal–regulated protein kinase 2
KSR: kinase suppressor of Ras
MD: molecular dynamics
PCA: principal component analysis
PDK1: 3-phosphoinositide-dependent protein kinase 1
PKA: cAMP-dependent protein kinase
R-spine: regulatory spine
WT-C: wild-type PKA catalytic subunit
