# Supplementary figures and images for: Decoding the Interactions Regulating the Active State Mechanics of Eukaryotic Protein Kinases
Source: PLoS Biol. 2016 Nov 30;14(11):e2000127. doi: 10.1371/journal.pbio.2000127 (PMC5130182; doi:10.1371/journal.pbio.2000127)

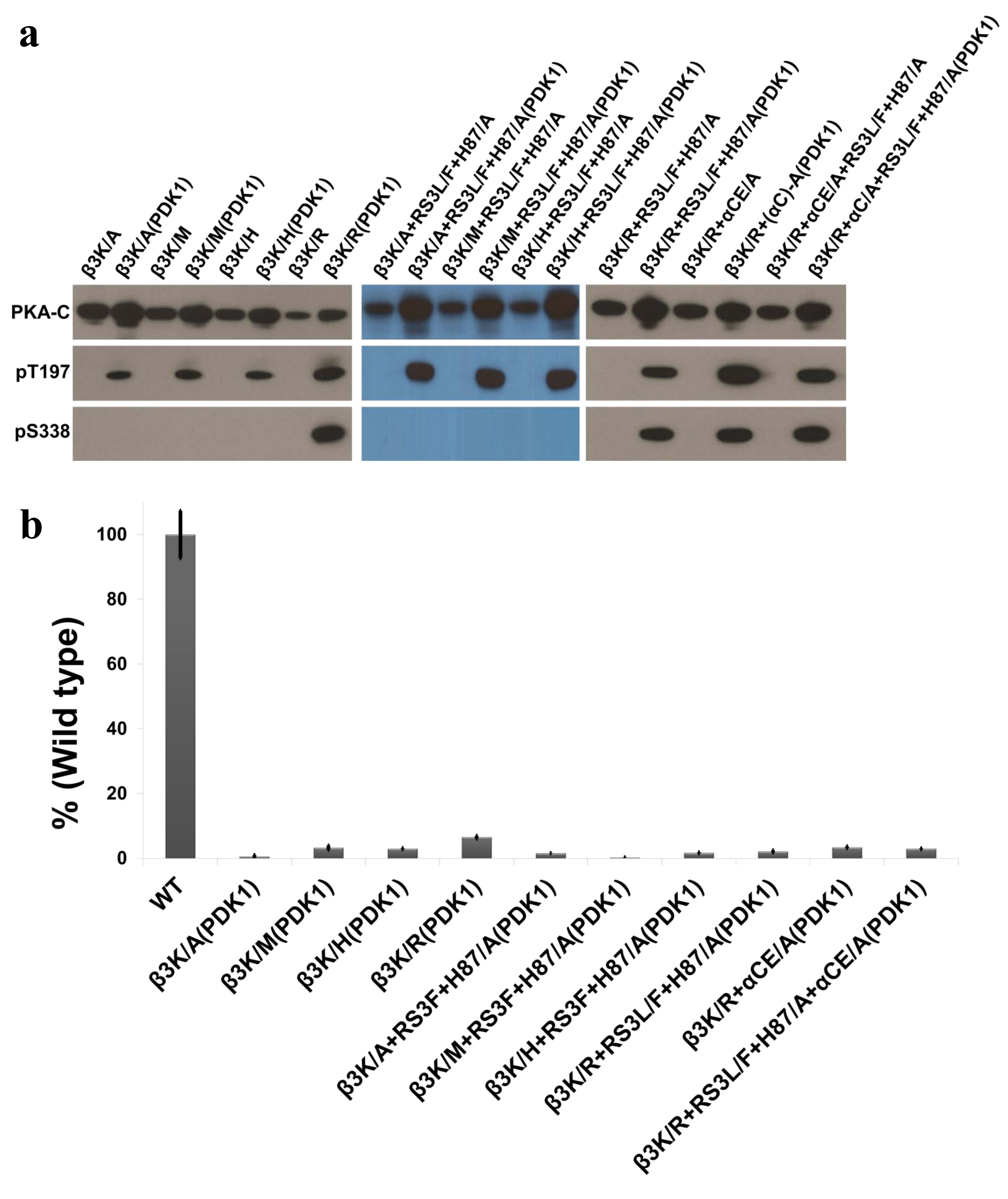

Supplement: S1 Fig — A. Western blot of the different mutants showing the expression (PKA-C) and autophosphorylation state of the AL and C-tail (pT197 and pS338, respectively). B. Level of radioactive phosphoryl transfer of the different mutants as compared to the WT-C data represents the mean and standard deviation shown in S1 Table. (TIF) [file pbio.2000127.s001.tif]

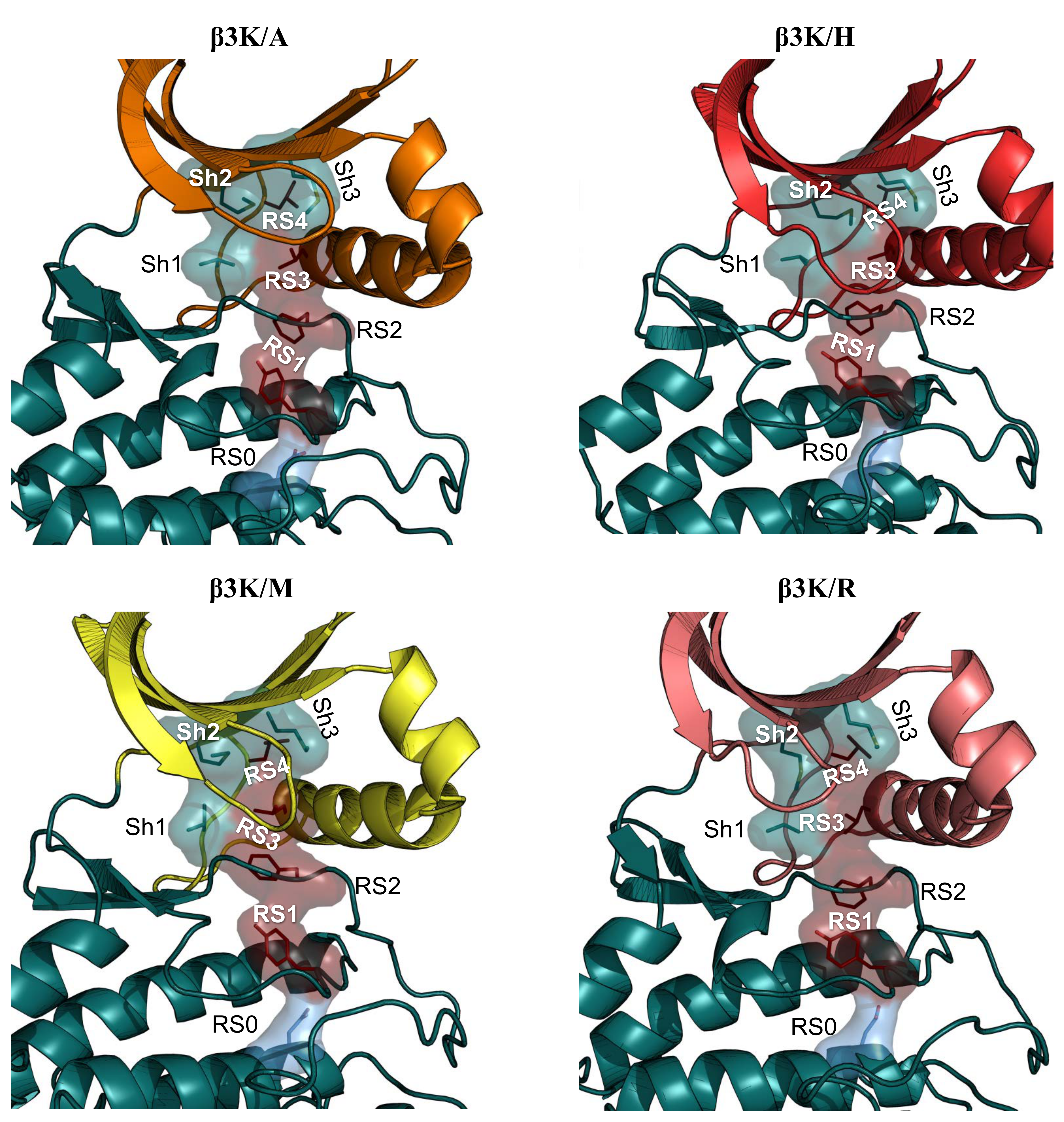

Supplement: S3 Fig — Structures of β3K/A (orange and teal), β3K/M (yellow and teal), β3K/H (red and teal), and β3K/R (pink and teal) are aligned with the WT-C (grey and olive) intermediate conformation. (TIF) [file pbio.2000127.s003.tif]

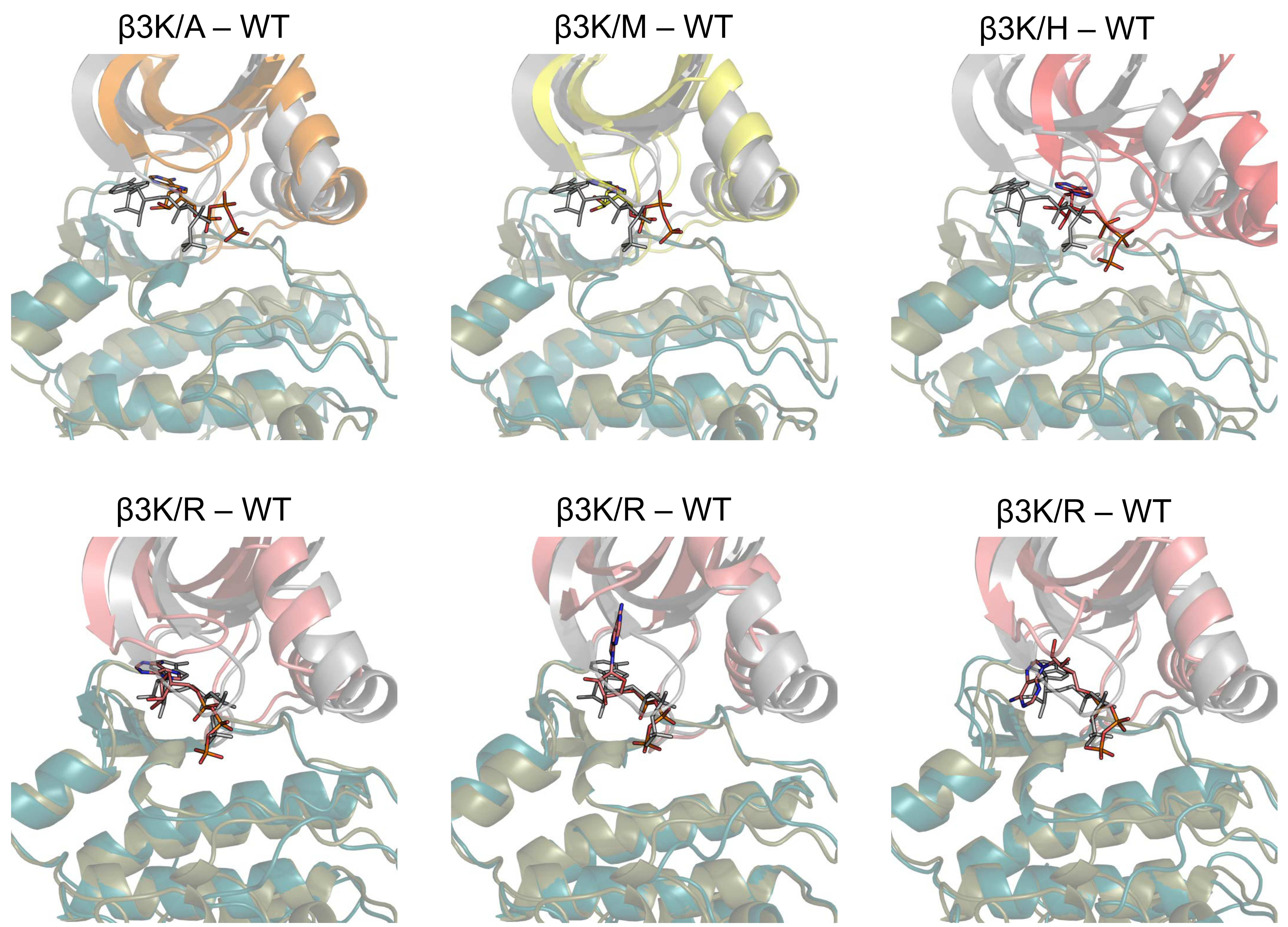

Supplement: S6 Fig — In comparison to the WT-C, structures of β3K/A (orange and teal), β3K/M (yellow and teal), β3K/H (red and teal), and β3K/R (pink and teal) are aligned with the WT-C (grey and olive) intermediate conformation. The different ATP conformations illustrated for the β3K/R mutant are snapshots at approximately 220 ns, 500 ns, and 700ns. (TIF) [file pbio.2000127.s006.tif]
