# Supplementary material for: Decoding the Interactions Regulating the Active State Mechanics of Eukaryotic Protein Kinases
Source: PLoS Biol. 2016 Nov 30;14(11):e2000127. doi: 10.1371/journal.pbio.2000127 (PMC5130182; doi:10.1371/journal.pbio.2000127)
Supplement: S1 Table — (PDF) [file pbio.2000127.s007.pdf]

	Activity (%WT)	Relative Standard Deviation
**WT**	100.00	7.36
**αCE/A**	0.40	0.10
**αCE/A(PDK1)**	12.85	1.30
**αCE/A+RS3L/F(PDK1)**	44.64	7.24
**αCE/A+H87/A(PDK1)**	42.21	5.99
**αCE/A+RS3L/F+H87/A(PDK1)**	197.1	19.71
**H87/A**	267.1	1.30
**RS3L/F**	31.85	4.84
**RS3L/F+H87/A**	3.91	0.35
**αCE/A+RS3L/F+H87/A+Sh2M/A+Sh3M/A(PDK1)**	3.45	0.02
**Sh2M/A+Sh3M/A(PDK1)**	117.9	9.28
**Sh2M/A+Sh3M/A+H87/A(PDK1)**	2.36	0.54
**Sh2M/A+Sh3M/A+H87/A+RS3L/F(PDK1)**	2.36	0.54
**Sh2M/A+Sh3M/A+RS3L/A(PDK1)**	101.4	6.37
**Sh2M/A+Sh3M/A+Sh1V/G(PDK1)**	1.17	0.73
**β3K/A(PDK1)**	0.51	0.52
**β3K/M(PDK1)**	3.25	0.70
**β3K/H(PDK1)**	2.94	0.22
**β3K/R(PDK1)**	6.51	0.46
**β3K/A+RS3F+H87/A(PDK1)**	1.59	0.07
**β3K/M+RS3F+H87/A(PDK1)**	0.20	0.11
**β3K/H+RS3F+H87/A(PDK1)**	1.67	0.20
**β3K/R+RS3L/F+H87/A(PDK1)**	2.10	0.34
**β3K/R+αCE/A(PDK1)**	3.37	0.36
**β3K/R+RS3L/F+H87/A+αCE/A(PDK1)**	2.92	0.18
**β3K/A+(GL)G/K**	424.9	19.58
**β3K/M+(GL)G/K(PDK1)**	1.84	0.12
**β3K/H+(GL)G/K(PDK1)**	2.53	0.04
**(GL)G/M**	114.8	10.2
