# Supplementary material for: Decoding the Interactions Regulating the Active State Mechanics of Eukaryotic Protein Kinases
Source: PLoS Biol. 2016 Nov 30;14(11):e2000127. doi: 10.1371/journal.pbio.2000127 (PMC5130182; doi:10.1371/journal.pbio.2000127)
Supplement: S2 Table — (PDF) [file pbio.2000127.s008.pdf]

Structure	Distance (Å)	
**WT-Open (G-loop - αF-helix)**	26.2	
**WT-Open (αC-helix- αF-helix)**	21.6	
**WT-Intermediate (G-loop - αF-helix)**	22.8	
**WT-Intermediate (αC-helix- αF-helix)**	20.7	
**WT-Closed (G-loop - αF-helix)**	19.3	
**WT-Closed (αC-helix- αF-helix)**	19.7	
**MD Simulation**	**Average distance (Å)**	**Standard Deviation**
**WT (G-loop - αF-helix)**	22.2	1.4
**WT (αC-helix- αF-helix)**	20.6	0.6
**β3K/A (G-loop - αF-helix)**	22.3	1.3
**β3K/A (αC-helix- αF-helix)**	21.2	0.6
**β3K/M (G-loop - αF-helix)**	21.1	0.6
**β3K/M (αC-helix- αF-helix)**	21.1	0.5
**β3K/H (G-loop - αF-helix)**	19.2	0.5
**β3K/H (αC-helix- αF-helix)**	20.1	0.5
**β3K/R (G-loop - αF-helix)**	25.8	1.4
**β3K/R (αC-helix- αF-helix)**	20.4	0.3
