# Supplementary material for: Decoding the Interactions Regulating the Active State Mechanics of Eukaryotic Protein Kinases
Source: PLoS Biol. 2016 Nov 30;14(11):e2000127. doi: 10.1371/journal.pbio.2000127 (PMC5130182; doi:10.1371/journal.pbio.2000127)
Supplement: S3 Table — (PDF) [file pbio.2000127.s009.pdf]

RESIDUE	β3K/A	β3K/M	β3K/H	β3K/R
LEU40	0.003471	N/A	0.003114	0.016423
ASP41	N/A	N/A	N/A	N/A
GLN42	0.03741	0.002832	0.250098	0.237241
PHE43	0.023685	0.000777	0.057622	0.076039
ASP44	0.000519	N/A	N/A	N/A
ARG45	0.000118	N/A	0.004973	0.001476
ILE46	0.011494	N/A	0.049967	0.050197
LYS47	0.047867	0.052462	0.040967	0.140166
THR48	0.05202	0.009227	N/A	0.04273
LEU49	0.113381	0.159448	0.064652	0.008117
GLY50	N/A	N/A	N/A	N/A
THR51	N/A	N/A	0.322166	N/A
GLY52	0.021904	1.07655	0.010946	0.160484
SER53	N/A	N/A	0.190499	N/A
PHE54	0.373385	1.021503	0.399246	1.010035
GLY55	0.021062	1.11274	0.114869	N/A
ARG56	0.017274	0.302215	N/A	N/A
VAL57	0.083382	0.233763	0.09526	0.047015
MET58	N/A	0.003562	0.017097	N/A
LEU59	N/A	N/A	N/A	N/A
VAL60	N/A	N/A	N/A	N/A
LYS61	N/A	N/A	0.308582	0.237072
HIS62	0.014516	0.000787	3.023511	3.122463
LYS63	0.000423	N/A	0.243439	0.242465
GLU64	N/A	N/A	0.889087	0.943508
SER65	N/A	N/A	0.714579	0.763834
GLY66	N/A	N/A	0.046359	0.037011
ASN67	0.001829	0.033058	0.116433	0.083002
HIS68	0.090359	N/A	1.156718	0.801974
TYR69	0.005061	0.017685	0.000845	0.002823
ALA70	0.002676	0.01432	0.006037	0.001902
MET71	0.034465	0.084128	0.033413	0.017578
LYS72	0.045057	1.65864	3.605534	0.991078
ILE73	0.153935	N/A	N/A	N/A
LEU74	0.027116	0.053229	N/A	0.009967
ASP75	N/A	0.012687	0.256145	N/A
LYS76	0.022095	N/A	0.011601	0.000768
GLN77	N/A	N/A	N/A	N/A
LYS78	N/A	0.004614	N/A	0.005738
VAL79	N/A	N/A	N/A	N/A
VAL80	0.295339	N/A	N/A	N/A
LYS81	0.454584	0.002496	0.001164	0.002982
LEU82	0.293722	N/A	N/A	N/A
LYS83	0.872175	N/A	N/A	N/A
GLN84	N/A	N/A	N/A	N/A
ILE85	0.055198	N/A	N/A	N/A
GLU86	0.003019	N/A	N/A	N/A
HIS87	1.784169	1.908534	0.178896	0.320217

**THR88**	0.039661	0.003225	0.001565	0.004081
**LEU89**	0.045319	0.00036	0.017258	0.001949
**ASN90**	0.123904	0.022526	0.806196	N/A
**GLU91**	2.04168	2.097057	0.004055	0.112411
**LYS92**	0.474477	0.029778	0.025245	0.010097
**ARG93**	0.122076	0.00173	0.550021	0.04074
**ILE94**	0.452173	0.169483	0.033679	0.008501
**LEU95**	0.007931	0.07183	0.01657	0.08483
**GLN96**	0.024897	N/A	0.037128	N/A
**ALA97**	N/A	N/A	N/A	N/A
**VAL98**	0.004889	N/A	0.001556	0.000906
**ASN99**	0.000426	0.057822	0.030117	N/A
**PHE100**	0.011889	0.010244	0.036061	0.015345
**PRO101**	0.014708	0.014251	N/A	0.009263
**PHE102**	N/A	N/A	0.004747	0.029227
**LEU103**	0.009437	0.003852	N/A	0.056317
**VAL104**	N/A	N/A	N/A	N/A
**LYS105**	N/A	0.04879	0.023321	0.007584
**LEU106**	0.095947	0.036673	0.02282	0.101822
**GLU107**	N/A	N/A	N/A	N/A
**PHE108**	N/A	N/A	N/A	N/A
**SER109**	0.165569	N/A	0.015068	0.060519
**PHE110**	0.129315	N/A	N/A	0.028264
**LYS111**	0.024887	0.002581	N/A	0.002093
**ASP112**	N/A	N/A	N/A	N/A
**ASN113**	N/A	N/A	N/A	N/A
**SER114**	N/A	0.017986	0.021818	0.048367
**ASN115**	N/A	N/A	N/A	N/A
**LEU116**	0.011413	0.018546	0.031958	0.015637
**TYR117**	0.00443	N/A	N/A	0.00366
**MET118**	0.352393	N/A	0.090012	N/A
**VAL119**	0.019603	0.077068	0.004389	N/A
**MET120**	N/A	0.041334	N/A	N/A
**GLU121**	N/A	0.009716	N/A	N/A
**TYR122**	N/A	0.208779	0.318172	0.034314
**VAL123**	N/A	N/A	N/A	N/A
**ALA124**	N/A	N/A	N/A	0.316887
**GLY125**	0.02199	N/A	0.214839	1.293193
**GLY126**	N/A	0.076559	0.107926	N/A
**GLU127**	N/A	N/A	N/A	0.021216
**MET128**	0.0487	0.300476	N/A	0.513097
**PHE129**	N/A	N/A	N/A	N/A
**SER130**	N/A	N/A	N/A	N/A
**HIS131**	0.753332	0.031648	0.003247	0.108828
**LEU132**	0.166602	0.049203	0.018906	N/A
**ARG133**	0.049326	0.078135	N/A	N/A
**ARG134**	0.02659	0.000202	0.020166	0.000842
**ILE135**	0.027904	0.087729	0.026213	0.022661
**GLY136**	N/A	0.106339	N/A	N/A

**ARG137**	1.176353	1.810243	1.143096	0.242006
**PHE138**	0.284517	0.751627	0.320305	0.72335
**SER139**	1.384343	0.049903	0.009728	1.528237
**GLU140**	0.025946	0.010347	0.000978	0.008774
**PRO141**	0.016967	0.023015	N/A	0.007811
**HIS142**	0.474607	N/A	0.484717	0.319112
**ALA143**	0.000512	N/A	N/A	N/A
**ARG144**	0.069436	0.013439	0.004442	0.018122
**PHE145**	0.004452	N/A	0.002805	N/A
**TYR146**	0.198805	0.201057	0.294141	0.223537
**ALA147**	0.004273	0.002155	0.016765	0.006788
**ALA148**	0.000156	N/A	0.020532	0.01159
**GLN149**	N/A	N/A	0.049529	0.177832
**ILE150**	0.006315	0.015117	0.218776	0.041609
**VAL151**	0.104883	0.264589	0.164414	0.096654
**LEU152**	0.009993	0.021563	0.067724	0.01377
**THR153**	0.023558	0.019378	0.005915	0.003038
**PHE154**	0.027082	0.07346	0.194663	0.068024
**GLU155**	0.132451	0.078044	0.067818	0.037711
**TYR156**	0.005593	0.004713	0.126824	0.088819
**LEU157**	0.00776	N/A	0.012543	0.047209
**HIS158**	0.063934	0.080705	4.83325	5.11204
**SER159**	N/A	N/A	0.082839	0.005412
**LEU160**	0.01	0.003316	0.079058	0.049542
**ASP161**	0.018289	0.001502	0.033566	0.04412
**LEU162**	N/A	0.00574	0.200175	0.20624
**ILE163**	0.068121	0.025629	0.066239	0.101187
**TYR164**	0.589612	0.649563	N/A	N/A
**ARG165**	0.850468	1.715101	N/A	0.495224
**ASP166**	0.477388	1.115199	N/A	0.244793
**LEU167**	0.993179	1.074897	N/A	N/A
**LYS168**	1.114318	0.27541	N/A	0.111257
**PRO169**	N/A	N/A	N/A	N/A
**GLU170**	0.626898	0.874388	0.033211	0.258043
**ASN171**	0.694811	0.522816	0.174806	1.089908
**LEU172**	0.027251	0.244271	0.063047	0.07856
**LEU173**	N/A	0.00838	N/A	N/A
**ILE174**	N/A	N/A	N/A	N/A
**ASP175**	0.403623	0.59562	0.348706	0.278769
**GLN176**	0.206657	0.375636	0.050576	N/A
**GLN177**	0.275881	0.978237	1.115738	0.382418
**GLY178**	0.026002	0.020685	0.033135	0.016774
**TYR179**	0.095092	0.033778	0.007529	N/A
**ILE180**	0.326703	N/A	N/A	N/A
**GLN181**	0.028543	0.035859	N/A	0.012667
**VAL182**	N/A	N/A	N/A	N/A
**THR183**	0.53156	0.11348	0.351283	N/A
**ASP184**	4.211661	3.625248	0.78601	0.494194
**PHE185**	0.655994	0.441579	0.200431	0.279175

**GLY186**	0.743847	0.772856	N/A	0.664901
**PHE187**	1.041935	1.556154	0.139114	0.439299
**ALA188**	0.001281	N/A	N/A	0.007537
**LYS189**	0.030949	0.168163	0.02461	0.039483
**ARG190**	0.071412	0.067851	0.022303	0.0112
**VAL191**	0.042087	0.118372	0.031237	0.104494
**LYS192**	N/A	0.030619	0.001686	0.066678
**GLY193**	0.029678	0.188666	N/A	0.080272
**ARG194**	0.022444	0.061365	0.2548	0.006959
**THR195**	0.12612	0.064454	0.000481	N/A
**TRP196**	0.033831	0.006665	N/A	0.072726
**T2P197**	0.021531	0.115643	N/A	0.257924
**LEU198**	0.457649	0.493751	0.022098	3.609593
**CYM199**	0.87686	0.469671	0.04123	0.164058
**GLY200**	1.785542	0.402633	0.126905	1.156706
**THR201**	0.229975	0.282344	0.081466	0.862691
**PRO202**	0.072009	0.042777	N/A	0.110169
**GLU203**	N/A	N/A	N/A	0.127
**TYR204**	0.155557	0.167693	0.222244	0.029008
**LEU205**	0.183461	0.196972	0.063724	0.587928
**ALA206**	0.000352	N/A	N/A	N/A
**PRO207**	0.002381	N/A	0.001971	0.03412
**GLU208**	N/A	0.004654	0.002229	0.028283
**ILE209**	0.056625	0.037277	6.23E-05	0.045542
**ILE210**	0.131242	0.016964	N/A	0.017739
**LEU211**	0.14962	0.002213	0.124548	0.361434
**SER212**	N/A	N/A	N/A	0.111061
**LYS213**	0.124092	N/A	N/A	0.63465
**GLY214**	0.215404	N/A	N/A	0.6218
**TYR215**	0.159816	0.009038	0.00255	0.003813
**ASN216**	0.672239	0.750645	0.154622	1.637737
**LYS217**	0.006525	0.081815	0.069395	0.042906
**ALA218**	N/A	N/A	N/A	N/A
**VAL219**	N/A	0.293039	N/A	N/A
**ASP220**	0.050777	0.367353	0.131053	0.121872
**TRP221**	0.770598	0.344331	0.288721	0.098385
**TRP222**	N/A	N/A	0.002199	0.007922
**ALA223**	0.016083	0.04636	0.030346	0.120554
**LEU224**	0.01033	0.021748	0.024704	N/A
**GLY225**	N/A	0.004864	0.007192	0.013625
**VAL226**	0.080626	N/A	N/A	0.003857
**LEU227**	0.005792	0.041761	0.036189	N/A
**ILE228**	0.049245	0.027066	0.005722	0.010839
**TYR229**	0.084225	0.190068	0.133887	0.233036
**GLU230**	N/A	0.139972	N/A	0.049426
**MET231**	0.026127	0.108436	0.037291	0.022604
**ALA232**	0.003058	0.168369	0.137924	0.05065
**ALA233**	0.123274	0.301467	0.114617	0.180982
**GLY234**	0.114979	0.161957	0.125755	0.122186

**TYR235**	0.051259	0.336847	0.02894	0.263334
**PRO236**	N/A	0.018593	N/A	N/A
**PRO237**	0.011051	0.153718	0.002234	0.071138
**PHE238**	0.026224	0.090922	0.007133	0.060178
**PHE239**	0.005234	0.114963	0.456218	0.083156
**ALA240**	N/A	0.018091	0.034084	0.030294
**ASP241**	0.028097	0.091888	0.100416	0.087653
**GLN242**	N/A	0.004801	0.097043	0.013303
**PRO243**	0.000697	N/A	0.008975	0.000832
**ILE244**	0.011409	0.014306	0.065327	0.040586
**GLN245**	0.037197	0.074109	0.071546	0.07392
**ILE246**	N/A	0.011019	N/A	N/A
**TYR247**	0.084056	0.063851	0.040341	0.290133
**GLU248**	0.006192	0.007966	0.014906	0.014374
**LYS249**	0.018001	0.074788	0.048056	0.024589
**ILE250**	0.011351	N/A	0.128042	0.001057
**VAL251**	0.022183	N/A	0.116735	N/A
**SER252**	0.421939	0.016855	0.451031	0.016548
**GLY253**	0.389696	0.053363	0.766256	0.050073
**LYS254**	0.290725	N/A	0.505496	0.051785
**VAL255**	0.073512	0.038628	0.13659	0.042637
**ARG256**	0.010889	0.027854	0.006642	0.008803
**PHE257**	N/A	0.084439	N/A	0.051753
**PRO258**	0.000184	0.037557	0.014335	0.061647
**SER259**	0.001068	0.00159	0.017127	0.020207
**HIS260**	0.084698	0.028929	1.331353	1.257452
**PHE261**	0.027178	0.026698	0.045452	0.115587
**SER262**	0.049817	0.00113	0.017715	0.014361
**SER263**	0.001021	0.000349	0.003572	0.000423
**ASP264**	0.091018	N/A	0.039369	0.041004
**LEU265**	0.006696	0.003034	0.062709	0.097242
**LYS266**	N/A	N/A	0.005544	0.015254
**ASP267**	N/A	0.000962	0.467456	0.447478
**LEU268**	0.037752	0.019905	0.09689	0.101441
**LEU269**	0.046839	0.046711	0.002184	N/A
**ARG270**	0.005699	0.000234	0.046248	0.029521
**ASN271**	0.315294	0.283227	0.351299	0.365455
**LEU272**	0.161306	0.408909	0.076208	0.193635
**LEU273**	0.019491	0.084818	0.045492	0.044426
**GLN274**	0.045384	0.037813	N/A	N/A
**VAL275**	N/A	N/A	N/A	N/A
**ASP276**	0.047171	0.043393	0.06546	0.035672
**LEU277**	N/A	N/A	N/A	N/A
**THR278**	0.072173	0.033363	0.018209	0.001533
**LYS279**	0.03368	0.000821	0.032983	0.008466
**ARG280**	0.003213	0.003809	N/A	N/A
**PHE281**	1.118366	2.391037	0.607569	0.46524
**GLY282**	0.525081	0.831141	1.266761	0.566732
**ASN283**	0.058088	0.282757	0.456521	0.142196

**LEU284**	0.870549	0.471288	1.343617	0.100826
**LYS285**	0.283284	0.144921	0.844568	0.34127
**ASN286**	0.237811	0.072955	0.307075	0.263676
**GLY287**	N/A	N/A	0.175633	N/A
**VAL288**	0.598084	0.3528	0.578864	0.105885
**ASN289**	2.765844	1.81117	0.108166	0.038026
**ASP290**	0.537444	0.130866	0.136731	0.148725
**ILE291**	4.048579	2.712439	0.035322	0.041221
**LYS292**	0.94955	0.386528	0.240104	0.055326
**ASN293**	0.308399	0.24152	0.249307	0.225142
**HIS294**	0.182201	0.071419	3.037147	3.017592
**LYS295**	0.049284	0.017622	0.220636	0.113813
**TRP296**	0.00988	0.00179	0.146149	0.147709
**PHE297**	0.011993	0.038703	0.035943	0.048594
**ALA298**	N/A	N/A	N/A	N/A
**THR299**	0.024712	0.020018	0.022269	0.015175
**THR300**	0.004344	0.010471	0.021584	0.013693
