# Supplementary material for: Decoding the Interactions Regulating the Active State Mechanics of Eukaryotic Protein Kinases
Source: PLoS Biol. 2016 Nov 30;14(11):e2000127. doi: 10.1371/journal.pbio.2000127 (PMC5130182; doi:10.1371/journal.pbio.2000127)
Supplement: S4 Table — (PDF) [file pbio.2000127.s010.pdf]

		WT-S		WT-MD	β3K/A	β3K/M	β3K/H	β3K/R
**ATP-$Mg^{2+}_{(1)}$**	**Distance (Å)**	2.1	**Average Distance (Å)**	1.9	1.9	1.9	1.9	1.9
			**Standard Deviation**	0.1	0.1	0.0	0.1	0.0
**ATP-$Mg^{2+}_{(2)}$**	**Distance (Å)**	2.1	**Average Distance (Å)**	1.8	1.9	1.9	1.8	1.9
			**Standard Deviation**	0.0	0.0	0.0	0.0	0.0
**ATP-E121**	**Distance (Å)**	2.9	**Average Distance (Å)**	4.2	3.1	2.9	3.0	10.6
			**Standard Deviation**	1.5	0.7	0.2	0.2	5.4
**ATP-β3K**	**Distance (Å)**	2.8	**Average Distance (Å)**	1.9	5.0	2.6	2.5	2.9
			**Standard Deviation**	0.3	0.6	0.3	0.6	1.3
**D184-$Mg2^{+}_{(1)}$**	**Distance (Å)**	2.3	**Average Distance (Å)**	1.9	1.9	1.9	1.9	1.9
			**Standard Deviation**	0.1	0.1	0.1	0.1	0.1
**D184-$Mg2^{+}_{(2)}$**	**Distance (Å)**	2.2	**Average Distance (Å)**	1.9	1.9	1.9	1.9	1.9
			**Standard Deviation**	0.1	0.0	0.0	0.1	0.1
**N171-$Mg2^{+}_{(2)}$**	**Distance (Å)**	2.1	**Average Distance (Å)**	2.0	3.7	3.6	2.0	3.3
			**Standard Deviation**	0.1	1.6	1.0	0.1	1.3
**β3K-(αC)E**	**Distance (Å)**	2.8	**Average Distance (Å)**	2.8	6.5	4.5	3.2	2.4
			**Standard Deviation**	1.0	0.5	1.2	1.3	0.8
**pT197-H87**	**Distance (Å)**	3.7	**Average Distance (Å)**	3.7	8.2	7.7	2.2	9.2
			**Standard Deviation**	0.8	1.0	0.9	0.7	2.3
**αCE-$Mg2^{+}_{(1)}$**	**Distance (Å)**	5.5	**Average Distance (Å)**	5.0	1.9	2.0	6.1	7.0
			**Standard Deviation**	0.6	0.1	0.1	1.3	0.7
**H87-ATP**	**Distance (Å)**	9.5	**Average Distance (Å)**	8.5	2.2	2.1	8.4	3.9
			**Standard Deviation**	0.7	0.8	0.4	0.5	2.0
