# Supplementary material for: Decoding the Interactions Regulating the Active State Mechanics of Eukaryotic Protein Kinases
Source: PLoS Biol. 2016 Nov 30;14(11):e2000127. doi: 10.1371/journal.pbio.2000127 (PMC5130182; doi:10.1371/journal.pbio.2000127)
Supplement: S5 Table — (PDF) [file pbio.2000127.s011.pdf]

a

	Tm	Standard deviation
**WT (Apo)**	39.8	0.1
**WT (ATP)**	42.3	0.0
**WT (ATP+pseudo-substrate)**	43.3	0.1
**WT (pseudo-substrate)**	39.7	0.4
**β3K/A+(GL)G/K (Apo)**	35.9	0.0
**β3K/A+(GL)G/K (ATP)**	39.9	0.0
**β3K/A+(GL)G/K (ATP+pseudo-substrate)**	41.7	0.2
**β3K/A+(GL)G/K (pseudo-substrate)**	33.3	0.8
**β3K/M+(GL)G/K(PDK1) (Apo)**	36.8	0.2
**β3K/M+(GL)G/K(PDK1) (ATP)**	38.7	0.0
**β3K/M+(GL)G/K(PDK1) (ATP+pseudo-substrate)**	38.7	0.1
**β3K/M+(GL)G/K(PDK1) (pseudo-substrate)**	36.8	0.1
**β3K/H+(GL)G/K(PDK1) (Apo)**	35.3	0.1
**β3K/H+(GL)G/K(PDK1) (ATP)**	36.7	0.2
**β3K/H+(GL)G/K(PDK1) (ATP+pseudo-substrate)**	36.5	0.1
**β3K/H+(GL)G/K(PDK1) (pseudo-substrate)**	34.8	0.2
**β3K/R(PDK1) (Apo)**	35.7	0.1
**β3K/R(PDK1) (ATP)**	37.2	0.3
**β3K/R(PDK1) (ATP+pseudo-substrate)**	36.9	0.0
**β3K/R(PDK1) (pseudo-substrate)**	35.7	0.0
**β3K/A(PDK1) (Apo)**	37.7	0.3
**β3K/A(PDK1) (ATP)**	38.9	0.1
**β3K/A(PDK1) (ATP+pseudo-substrate)**	39.7	0.3
**β3K/A(PDK1) (pseudo-substrate)**	38.0	0.1

b

	WT	β3K/A+ (GL)G/K	β3K/M+(GL)G/K (PDK1)	β3K/H+(GL)G/K (PDK1)	β3K/R (PDK1)	β3K/A (PDK1)
**Tm(ATP)-Tm(Apo)**	2.5	3.94	1.93	1.415	1.43	1.2
**Relative Standard deviation**	0.15	0.05	0.2	0.305	0.38	0.4
**Tm(ATP+Substrate)-Tm(Apo)**	3.44	5.835	1.875	1.2	1.165	2.0
**Relative Standard deviation**	0.1	0.205	0.255	0.305	0.265	0.6
**Tm(Substrate)-Tm(Apo)**	-0.12	-2.66	-0.015	-0.5	-0.01	0.4
**Relative Standard deviation**	0.47	0.82	0.335	0.28	0.17	0.4
